# Ways to improve surgical outcomes in low- and middle-income countries

**DOI:** 10.2471/BLT.22.287950

**Published:** 2022-09-20

**Authors:** Cornelia Anne Barth, Andreas Wladis, Nobhojit Roy, Catherine Blake, Sanda Muhammad Kolo, Cliona O'Sullivan

**Affiliations:** a Cochrane Switzerland, Unisanté, Route de la Corniche 10, CH-1010, Lausanne, Switzerland.; b Biomedical and Clinical Sciences, Linköping University, Linköping, Sweden.; c The George Institute of Global Health, New Delhi, India.; d School of Public Health, Physiotherapy and Sports Science, University College Dublin, Dublin, Ireland.; e International Committee of the Red Cross, Maiduguri, Nigeria.

## Abstract

Global surgery initiatives such as the *Lancet* Commission on Global Surgery have highlighted the need for increased investment to enhance surgical capacity in low- and middle-income countries. A neglected issue, however, is surgery-related rehabilitation, which is known to optimize functional outcomes after surgery. Increased investment to enhance surgical capacity therefore needs to be complemented by promotion of rehabilitation interventions. We make the case for strengthening surgery-related rehabilitation in lower-resource countries, outlining the challenges but also potential solutions and policy directions. Proposed solutions include greater leadership and awareness, augmented by recent global efforts around the World Health Organization’s Rehabilitation 2030 initiative, and professionalization of the rehabilitation workforce. More research on rehabilitation is needed in low- and middle-income countries, along with support for system approaches, notably on strengthening and integrating rehabilitation within the health systems. Finally, we outline a set of policy implications and recommendations, aligned to the components of the national surgical plan proposed by the *Lancet* Commission: infrastructure, workforce, service delivery, financing, and information management. Collaboration and sustained efforts to embed rehabilitation within national surgical plans is key to optimize health outcomes for patients with surgical conditions and ensure progress towards sustainable development goal 3: health and well-being for all.

## Résumé

À l'instar de la Commission *Lancet* sur la chirurgie mondiale, des initiatives internationales consacrées à ce sujet ont mis en évidence le besoin d'investir davantage dans le renforcement des capacités chirurgicales dans les pays à revenu faible et intermédiaire. Néanmoins, la réadaptation post-chirurgicale, connue pour améliorer les résultats fonctionnels après une intervention, reste un enjeu largement ignoré. Ces investissements accrus visant à renforcer les capacités chirurgicales doivent donc aller de pair avec une promotion des services de réadaptation. Dans le présent document, nous plaidons pour le développement d'une réadaptation post-chirurgicale dans les pays à revenu faible et intermédiaire, en identifiant les défis mais aussi les orientations politiques et les solutions possibles. Parmi elles, un meilleur leadership et une prise de conscience, favorisée par les récents efforts mondiaux qui ont entouré l'initiative Réadaptation 2030 de l'Organisation mondiale de la Santé, ainsi qu'une professionnalisation du personnel dédié à la réadaptation. D'autres recherches sur la réadaptation sont nécessaires dans les pays à revenu faible et intermédiaire, tout comme l'apport d'un soutien aux approches systémiques, en particulier pour consolider et intégrer de telles pratiques dans les systèmes de santé. Enfin, nous dégageons une série de recommandations et d'implications politiques inspirés des éléments du plan chirurgical national proposé par la Commission *Lancet*: infrastructures, main-d'œuvre, prestations de services, financement et gestion des informations. La collaboration et la poursuite des efforts en vue d'inclure la réadaptation dans les plans chirurgicaux nationaux jouent un rôle crucial dans l'amélioration des résultats cliniques chez les patients souffrant de complications post-opératoires. En outre, elles permettront de progresser vers le troisième objectif de développement durable: santé et bien-être pour tous à tout âge.

## Resumen

Las iniciativas de cirugía a nivel mundial, como la Comisión *Lancet* sobre Cirugía Mundial, han destacado la necesidad de aumentar la inversión para mejorar la capacidad quirúrgica en los países de ingresos bajos y medios. Sin embargo, se ha descuidado la rehabilitación relacionada con la cirugía, que se sabe que optimiza los resultados funcionales después de la intervención. Por lo tanto, el incremento de la inversión para mejorar la capacidad quirúrgica se debe complementar con la promoción de intervenciones de rehabilitación. En este artículo se defiende la necesidad de reforzar la rehabilitación relacionada con la cirugía en los países con menos recursos, y se exponen los desafíos, pero también las posibles soluciones y orientaciones políticas. Las soluciones propuestas incluyen un mayor liderazgo y concienciación, potenciados por los recientes esfuerzos mundiales en torno a la iniciativa Rehabilitación 2030 de la Organización Mundial de la Salud, y la profesionalización del personal de rehabilitación. Se necesita más investigación sobre la rehabilitación en los países de ingresos bajos y medios, junto con el apoyo a los enfoques sistémicos, en particular sobre el fortalecimiento y la integración de la rehabilitación dentro de los sistemas sanitarios. Por último, se expone un conjunto de implicaciones y recomendaciones políticas, alineadas con los componentes del plan quirúrgico nacional que propone la Comisión *Lancet*: infraestructura, personal, prestación de servicios, financiación y gestión de la información. La colaboración y los esfuerzos sostenidos para integrar la rehabilitación en los planes quirúrgicos nacionales son fundamentales para optimizar los resultados sanitarios de los pacientes con afecciones quirúrgicas y asegurar el progreso hacia el tercer objetivo de desarrollo sostenible: salud y bienestar para todos.

## ملخص

سلطت المبادرات الجراحية العالمية مثل "لجنة لانسيت المعنية بالجراحة العالمية" الضوء على مدى الحاجة إلى ضخ استثمارات متزايدة لتحسين فرص توفير وإتاحة الخدمات الجراحية في الدول ذات الدخل المنخفض والدخل المتوسط. وبالرغم من ذلك، هناك قضية لم يتم التطرق إليها وهي عمليات إعادة التأهيل المرتبطة بالجراحات، والمعروف أنها تحقق أفضل النتائج الوظيفية بعد إجراء الجراحة. لذا، يجب أن تكون الزيادة في الاستثمارات الموجهة لتحسين فرص توفير وإتاحة الخدمات الجراحية مصحوبة بتعزيز وترقية التدخلات التأهيلية. نحن نتناول الأسباب التي تدفعنا لتقوية خدمات إعادة التأهيل المرتبطة بالجراحات في الدول شحيحة الموارد، ولن نبين التحديات فقط بل سنعرض الحلول المحتملة والتوجهات السائدة للسياسات. تتضمن الحلول المقترحة دورًا أكبر للقيادة ونشر الوعي وتعززها الجهود العالمية التي تُبذل مؤخرًا في "مبادرة إعادة التأهيل" لعام 2030 التي أطلقتها منظمة الصحة العالمية، علاوة على الإعداد المهني للقوى العاملة في مجال إعادة التأهيل. هناك حاجة لإجراء مزيد من الأبحاث بشأن إعادة التأهيل في الدول ذات الدخل المنخفض والدخل المتوسط جنبًا إلى جنب مع دعم نُهج النُظم، لا سيما الدعم الرامي إلى تقوية خدمات إعادة التأهيل ودمجها في النُظم الصحية. وفي النهاية، نوضح مجموعة من مقتضيات وتوصيات السياسات المتوافقة مع مكونات الخطة الجراحية الوطنية المقترحة من جانب "لجنة لانسيت": وهي البنية التحتية والقوى العاملة وتقديم الخدمات والتمويل وإدارة المعلومات. يكتسب كل من التعاون والجهود المستدامة الرامية لدمج إعادة التأهيل في الخطط الجراحية الوطنية أهمية بالغة من أجل تحقيق أفضل النتائج الصحية للمرضى ذوي الحالات التي تستدعي التدخل الجراحي وضمان إحراز تقدم باتجاه تحقيق هدف التنمية المستدامة رقم 3: "ضمان تمتّع الجميع بأنماط عيش صحية وبالرفاهية".

## 摘要

《柳叶刀》杂志全球外科委员会等组织发起的全球手术倡议中强调，需要增加投资以提高中低收入国家的手术能力。然而，与手术相关的康复治疗是一个被忽视的问题。众所周知，康复治疗可以改善术后的功能恢复效果。因此，需要推广康复干预，并将其作为为提高手术能力增加投资的补充。我们提出了强有力的理由来支持在资源匮乏国家加强与手术相关的康复治疗这一提议，并概述了面临的挑战以及潜在的解决方案和政策方向。提出的解决方案包括提高领导力和康复意识，吸引全球更多国家为世卫组织康复 2030 计划做出努力，以及建设专业的康复治疗人员队伍。中低收入国家需要开展更多康复治疗方面的研究，并为系统方法提供支持，特别是加强康复治疗和卫生系统的整合工作。最后，我们概述了一系列政策含义和建议，与《柳叶刀》杂志委员会提出的国家手术计划的内容框架（基础设施、劳动力、服务提供、融资和信息管理）保持一致。为将康复治疗纳入国家手术计划而展开的合作和持续努力是改善手术患者的健康护理效果和确保可持续发展目标 3（良好健康和福祉）取得进展的关键。

## Резюме

Глобальные инициативы в области хирургии, такие как Комиссия по глобальной хирургии *Lancet*, выявили необходимость увеличения инвестиций для укрепления хирургического потенциала в странах с низким и средним уровнем дохода. Однако при этом совершенно недостаточное внимание уделяется связанной с хирургическим вмешательством реабилитации, которая, как известно, оптимизирует функциональные результаты после такого вмешательства. Поэтому увеличение инвестиций в укрепление хирургического потенциала должно дополняться продвижением мероприятий по реабилитации. Авторы приводят доводы в пользу усиления реабилитации, связанной с хирургическим вмешательством, в странах с низким уровнем ресурсов, перечисляя задачи, а также потенциальные решения и направления политики. Предлагаемые решения включают в себя более активное руководство и осведомленность, дополненные недавними глобальными усилиями в рамках инициативы Всемирной организации здравоохранения «Реабилитация-2030», а также профессионализацию кадров в сфере реабилитационных услуг. Необходимы дополнительные исследования в области реабилитации в странах с низким и средним уровнем дохода, а также поддержка системных подходов, особенно по укреплению и интеграции реабилитации в системы здравоохранения. Наконец, авторы предлагают ряд политических последствий и рекомендаций, согласованных с компонентами национального хирургического плана, предложенного Комиссией *Lancet*: инфраструктурой, персоналом, предоставлением услуг, финансированием и управлением информацией. Сотрудничество и постоянные усилия по включению реабилитации в национальные хирургические планы имеют ключевое значение для оптимизации результатов лечения пациентов с хирургическими заболеваниями и обеспечения прогресса в достижении цели 3 в области устойчивого развития: здоровье и благополучие для всех.

## Introduction

The report of the *Lancet* Commission on Global Surgery 2030 highlights the shortage of essential, safe and affordable surgical procedures to more than half of the world’s population and the negative impact this shortage has on mortality, morbidity and functioning.^1^ The report calls for increased investment to enhance surgery and anaesthesia care within health systems in low- and middle-income countries so that at least 80% of the world’s population will have access to essential, safe, timely and affordable surgery by 2030. While we agree with the recommendations of the report, there is one critical element almost completely omitted: rehabilitation. 

Rehabilitation has been defined as “a set of interventions designed to optimize functioning and reduce disability in individuals with health conditions in interaction with their environment.”^2^ Functioning has been proposed as a third health indicator alongside mortality and morbidity.^3^ This description highlights the capacity of rehabilitation to enhance functional capacity, reduce morbidity and decrease mortality.^4^ An analysis of global estimates of the need for rehabilitation lists the prevalence of 25 health conditions that require rehabilitation.^5^ The authors state that in 2019, 2.41 billion people worldwide had conditions that would benefit from rehabilitation. Several of the most prevalent health conditions, such as orthopaedic and neurological injuries and cancers, also require surgery in combination with rehabilitation to achieve optimal functioning and reduce disability.

It is well-established that rehabilitation prevents complications and improves postoperative and long-term pain and functional outcomes across several health conditions.^6^^,^^7^ However, the role and effectiveness of rehabilitation extends further. Adequate rehabilitation can prevent or delay surgery in conditions such as low back pain, urinary incontinence or clubfoot^8^^–^^10^ and plays a fundamental role in injury prevention, such as prevention of falls.^11^ Prehabilitation is the element of rehabilitation that begins before surgery, such as endurance training or promotion of physical activity. Prehabilitation not only prepares for surgery, but also optimizes overall postoperative functional and health outcomes in conditions such as cancer and abdominal surgery.^12^^,^^13^ Furthermore, the timely combination of surgery and rehabilitation has the potential to enhance their mutual effects, as shown in, for example, spastic cerebral palsy, clubfoot, obstetric fistula and interventions after fracture.^14^^–^^17^

Without adequate rehabilitation, the outcome of many surgical procedures will be suboptimal. However, little attention is currently given to the importance of pre- and postoperative rehabilitation in low- and middle-income countries.^18^^,^^19^ We discuss the need to enhance and align rehabilitation capacity with surgical services in these countries to optimize outcomes for patients with conditions requiring surgery.

## Challenges

Strengthening surgery-related rehabilitation services in low- and middle-income countries is challenging.^18^ A chronic lack of investment in the rehabilitation workforce and in development of the sector in general means that rehabilitation is often not part of mainstream health services.^20^ The consequences for patients can be serious. Assistant staff who have been trained onsite or the patient’s relatives often struggle to provide informal care or any kind of rehabilitation, with scant supervision.^18^^,^^21^ There is often little awareness among surgeons and policy-makers about the need to advocate and ensure that rehabilitation is included in the planning of surgical services.^22^ The problem is compounded by a lack of evidence on surgery-related rehabilitation in lower-resource countries and in conflict settings. A recent systematic review on trauma and rehabilitation interventions emphasized the importance of timely post-surgery rehabilitation and reported a shortage of publications on the subject.^23^

Most research on rehabilitation has been conducted in high-income countries, where many factors – including patients’ health conditions and the types of surgery as well as the health system infrastructure and human resources – are not comparable to low- and middle-income countries. These differences are especially evident in conflict and emergency settings, where trauma surgery predominates.^24^^,^^25^ In high-income countries, rehabilitation is an integral part of usual care after surgery. Such care requires the necessary infrastructure, workforce and funding. Patient-related factors such as patients’ expectations and satisfaction need to be considered. Clinical studies in high-income countries tend to focus on comparisons of different types of structured rehabilitation programmes.^26^ These programmes and the evidence resulting from studies are of limited use and transferability to most low- and middle-income contexts.

The first step is to establish a general understanding of the processes and benefits of rehabilitation.^27^ Extended bed rest is still widespread practice in low- and middle-income countries, despite the risk of preventable complications due to the practice.^21^ Many trauma protocols in these countries are limited to acute medical care, thus overlooking the important role of early rehabilitation. The relevant training often involves only physicians and nurses rather than rehabilitation workers.^28^ While the importance of rehabilitation may be mentioned in clinical training, further guidance or evidence-based protocols are lacking.^29^

We approach the issue as physiotherapists and surgeons with long-term experience in lower-resource countries and humanitarian settings. Our own experiences confirm that surgery-related rehabilitation is rarely considered an essential part of the continuum of care.^30^^,^^31^ A study involving focus group discussions with physiotherapists from 18 low- and middle-income countries revealed the adverse effects of neglecting rehabilitation.^32^ Staffing posts for rehabilitation professionals in the public sector and decent employment conditions are rare.^33^ Even well-established education programmes within a country do not guarantee professional regulation and recognition of rehabilitation workers at health ministry level. Many rehabilitation professionals end up leaving their country. Others may work as non-clinicians for international organizations or in urban private facilities rather than alongside surgeons and other health professionals in public hospitals.^32^

The awareness of surgeons and other hospital staff in low- and middle-income countries is often limited to wound care after surgical intervention. To free up beds for new patients, the condition for discharge is commonly wound closure, after which advanced rehabilitation towards functional independence should start. A lack of rehabilitation facilities and follow-up in the form of accessible outpatient structures or community-based services often leads to this key stage of the rehabilitation process being left out.^18^ In conflict-affected settings the problem is exacerbated.^30^ Even simple aspects of a patient’s functioning, such as the need for secure transfers or mobility aids, are rarely considered when discharging patients from hospital. These shortcomings may be a result of rehabilitation not being systematically integrated within health systems and its potential not being sufficiently recognized. Patients find themselves at home, dependent on the help of others and at risk of developing further limitations on their functioning.^21^^,^^34^

## Solutions

### Leadership

Several recent global initiatives such as the World Health Organization’s (WHO) Emergency Medical Teams and Rehabilitation 2030 initiative are welcome efforts to raise awareness among policy decision-makers about developing rehabilitation capacity.^2^^,^^35^ In addition to these high-level efforts, leadership and commitment must also come from surgeons and rehabilitation professionals, patient groups, professional associations and other organizations active in their respective countries. The impact of Benin’s successful rehabilitation sector across West Africa, for example, is the result of such multistakeholder collaborations.^36^

We believe that multisectoral and multilevel leadership will yield several benefits: a unified voice and greater collaboration among the global rehabilitation community; improved awareness and advocacy of the importance of rehabilitation; a strategy for expanding the rehabilitation workforce; and the momentum to drive research that will inform policy. Together, these benefits will ultimately improve access to quality rehabilitation to meet population needs.

### Rehabilitation workforce

Strengthening the rehabilitation health workforce in contexts where existing staffing levels are low and where demand is greatest is a challenge.^2^^,^^32^^,^^33^^,^^37^ Investment is needed. The potential for delivery of rehabilitation training programmes through academic partnerships between high-income and low- and middle-income countries^38^ and between institutions within low- and middle-income countries (south–south partnerships) is promising. Another example is provided by a collaboration between university hospitals in Sweden and Zimbabwe to optimize rehabilitation outcomes after hand surgery. Such surgery is a medical field where functional outcome and socioeconomic reintegration are highly dependent on the quality of rehabilitation.^39^

Multisectoral collaboration is a key driver of knowledge translation from research into practice and strengthening of the rehabilitation workforce. *Early rehabilitation in conflicts and disasters* is a handbook for rehabilitation professionals working in conflict and disaster response and is the result of a joint project of humanitarian, professional and disease-specific organizations and individual experts from high- and lower-income countries.^40^ The book, which is freely available, could serve as a basis for development of contextualized courses and guidelines for low- and middle-income countries.

Where universities and nongovernmental organizations collaborate, practical experience in local settings and knowledge of training priorities can be combined with experience in curriculum development and education delivery. Establishing competency-based education programmes will allow population health needs to be matched to rehabilitation worker skill sets. These programmes can be informed by WHO’s Rehabilitation Competency Framework, a detailed framework that captures the key activities and competencies required to provide quality rehabilitation service delivery.^41^

Professionalization of the discipline is needed to enhance the professional qualities of the rehabilitation sector. This process requires increasing the number of people training and enhancing the quality of training programmes for rehabilitation professionals. Career pathways progressing from basic to advanced practice competencies are required. It may help to develop modular course structures beginning with training for mid-level rehabilitation workers and advancing to professional degrees in rehabilitation. Courses need to span rehabilitation practice for community- and primary care-based workers, up to specialized rehabilitation experts.

Online learning and educational technology can be harnessed to support these programmes and offer continuous professional development opportunities.^42^^,^^43^ The recent pandemic of coronavirus disease 2019 has accelerated innovation in and readiness for online learning. In some instances, the lack of a rehabilitation workforce has led to the development of novel care models that have proven successful. Examples include the deployment of community health workers providing surgery-related rehabilitation in primary and community health care and home-based rehabilitation programmes in Uganda.^44^ These initiatives can be further explored towards widespread use across different countries.

### Research

Research to inform best practice is essential for strengthening rehabilitation within health systems. Close collaboration between universities in high-income and low- and middle-income countries and with humanitarian and international cooperation agencies is necessary to merge research expertise with field experience, harness research funding opportunities and demonstrate impact.^39^ Several publications show that health research capacity-building in low- and middle-income countries requires further development and is necessary for advancing the quality of care in these countries. Equal partnership between collaborators in high- and lower-income countries is key, with patients and professionals from low- and middle-income countries leading the research agenda.^45^ Indeed, stakeholder involvement in research should be fostered to ensure that research priorities address population needs and to facilitate translation of research findings into practice. Participatory research approaches have proven useful in areas such as disability research and in humanitarian health programming.^46^^,^^47^ These approaches can be integrated and strengthened within the field of global surgery. A study on patients’ perspectives from Uganda illustrates the negative socioeconomic impact of injury and post-surgical disability in places where rehabilitation is not part of surgical care.^34^ Qualitative approaches are important for understanding the attitudes and perceptions towards rehabilitation among decision-makers such as surgeons or hospital managers and to identify and address potential barriers and facilitators.

Another research gap is quality evidence on the outcomes and impact of injury- and surgery-related rehabilitation in low- and middle-income settings.^29^ Establishing research partnerships and funding for multicentre, multicountry studies would create an evidence base for urgently needed rehabilitation programming. Studies need to be sufficiently statistically powered and complemented by health-systems research. Researchers have listed the many challenges of follow-up and surgical outcomes research in low- and middle-income countries, but make no mention of the role of rehabilitation.^48^ Follow-up challenges should be discussed and addressed among surgeons and rehabilitation professionals to improve data quality and research knowledge. Surgical data systems should always include rehabilitation data, such as functional and participatory outcomes, something which is currently missing in most low- and middle-income settings. Global surgery research projects have increased since the release of the *Lancet* Commission on Global Surgery report. Future research directions must also include the impact of rehabilitation on surgical outcomes.

### System approaches

Strengthening of rehabilitation should not be limited to health systems. Health ministries, higher education, social affairs and labour should join forces to allow professional regulation, education and training, decent working conditions, better infrastructure, adequate pay and retention of staff.^32^ Embedding rehabilitation professionals within a country’s health system would allow them to develop their potential and ensure teamwork with surgeons and other health professionals.^49^ This multidisciplinary collaboration needs to be prepared by integrating modules on surgery-related rehabilitation into the curricula of other medical professions, notably future surgeons.

Rehabilitation is often associated with disability in low- and middle-income countries. The strength of the sector is thus its position at the intersection of the health and social care professions, with the potential to advocate with both sectors. Rehabilitation as a health service leads to better functioning for patients; as a social service it prepares people with disabilities for enhanced social and economic participation within their communities. Rehabilitation must be seen as essential throughout the continuum of care, from health promotion and prevention, pre- and post-surgical rehabilitation, through non-surgical related interventions, up to the social inclusion of people with disabilities. Collaborations on a systemic level with the health, education and labour sectors are crucial to allow working towards a common vision: the optimal functioning of individuals with health conditions and thus of communities and society.

## Policy implications and recommendations

Achieving universal health coverage (UHC) – so that all people and communities receive the health services that they need without suffering financial hardship – is a key policy goal of the sustainable development goal agenda.^2^ Rehabilitation is mentioned as part of the full spectrum of essential, quality health services. To work towards UHC it is therefore essential that rehabilitation is included in policy development for surgical care where appropriate. The *Lancet* Commission strongly advocates for the development of national surgical plans to ensure “proper planning of surgical care delivery, education and research.” Yet rehabilitation, which is a key part of the continuum of care following some types of surgery, was not included.

To make progress in the development of surgery-related rehabilitation in low- and middle-income countries, there is a need for greater collaboration between surgery and rehabilitation professionals, and across the health, education and labour sectors. Rehabilitation needs to be integrated into national surgery policies. Below we outline some strategies to achieve this, using the national surgical plan framework: infrastructure, workforce, service delivery, financing and information management.^1^

### Infrastructure and products

Surgical facilities must be equipped to enable delivery of basic post-surgical rehabilitation interventions: for example, space to facilitate early mobilization and ambulation, chairs for upright sitting or assistive devices such as mobility aids. The continuum of care after surgery should include, where appropriate, referral systems to step-down facilities offering rehabilitation or to outpatient services to ensure that patients have access to essential rehabilitation services such as fitting of prosthetics.

### Workforce 

Education and training strategies within national surgical plans must include training of rehabilitation staff based on the population and needs of the country. Inclusion of rehabilitation within surgical training programmes is needed. WHO’s Rehabilitation Competency Framework^41^ can serve as a platform for educational institutions to design education and training programmes and to facilitate discussion among the education, labour and health sectors. Collaboration across these three sectors is crucial to ensure that graduates from rehabilitation programmes are recognized and eligible for employment within national health systems.

### Service delivery

Evidence-based surgical rehabilitation protocols need to be developed and adapted at the local level for the health conditions that would benefit most from rehabilitation. WHO is currently developing a package of interventions for rehabilitation which will serve as a platform to enable service providers to plan and implement specific rehabilitation interventions.^50^

### Financing 

Funding plans for basic surgical care pathways must include funding for the provision of rehabilitation interventions, including the necessary workforce, facilities and assistive devices. Of course, there are financial implications to providing surgery-related rehabilitation to those who need it. However, neglecting rehabilitation increases the chances of lengthy or permanent disability that has implications on individuals and households, such as the impact on return to work or schooling. In lower-resource settings, rehabilitation is considered a luxury and often available to patients based on ability to pay rather than need.

### Information management 

Surgical information systems need to include short- and longer-term patient outcomes relating to function (such as mobility and activities of daily living) and participation (such as health-related quality of life or return to work). These processes also need to be monitored to enhance quality improvement processes. Capacity-building for rehabilitation research must be championed and supported. Research priorities relating to rehabilitation that are locally relevant should be identified and funded.

## Conclusion

Surgery has the potential to save hundreds of thousands of lives, whether in peacetime or in conflicts. But mere survival is not good enough. We call on our colleagues in surgery and on managers and policy-makers in low- and middle-income countries to give surgery-related rehabilitation a greater role in their work towards achieving the targets of their national surgical plans. 
